# Pheochromocytoma Mimicking Acute Coronary Syndrome: A Case Report

**DOI:** 10.3389/fonc.2022.879714

**Published:** 2022-04-13

**Authors:** Yanwei Cheng, Lijie Qin, Long Chen

**Affiliations:** Department of Emergency, Henan Provincial People’s Hospital, People’s Hospital of Zhengzhou University, People’s Hospital of Henan University, Zhengzhou, China

**Keywords:** pheochromocytoma, catecholamine, acute coronary syndrome, electrocardiogram, case report

## Abstract

Pheochromocytoma is a rare catecholamine-secreting tumor with highly variable clinical presentations. We herein report a patient who presented to the emergency department with precordia pain, elevated myocardial enzymes, T-wave inversions on electrocardiogram and segmental ventricular wall motion abnormalities on echocardiography, which is normally managed as suspected acute coronary syndrome (ACS). However, the urgent coronary angiography showed normal coronary arteries. During his hospital stay, a sudden increase in blood pressure allowed us to suspect a pheochromocytoma, which was confirmed by elevated levels of catecholamines and by the finding of a right adrenal mass on magnetic resonance imaging. The tumor was successfully excised and the patient is now asymptomatic. This case illustrates that pheochromocytoma can present as a mimic of ACS but this is often difficult to diagnose at first glance and often misleads clinicians into making an incorrect diagnosis. In addition, clinicians should be familiar with clinical manifestations of pheochromocytoma, which can help raise clinical suspicion and facilitate the early diagnosis and treatment of pheochromocytoma.

## Introduction

Pheochromocytoma is a rare catecholamine-secreting neuroendocrine tumor that arises from chromaffin cells of the adrenal medulla or extra-adrenal paraganglia. Annually, only 2 to 9 per million of population are affected by pheochromocytoma (1). The clinical manifestations of pheochromocytoma varies widely, with fewer than 5% of patients presenting with the typical triad of symptoms consisting of headache, palpitations, and diaphoresis (2). Notably, when the paroxysmal excess release of catecholamines including norepinephrine and epinephrine into the circulation, pheochromocytoma can cause life-threatening cardiovascular complications and mimic acute coronary syndrome (ACS) *via* inducing severe vasoconstriction, coronary vasospasm, myocardial ischemia, injury, and necrosis (3), which often misleads clinicians, epically emergency physicians, into making an incorrect diagnosis.

Herein, we report the case of a 56-year-old male patient who presented with symptoms, elevated serum troponin, electrocardiographic and echocardiography changes suggestive of ACS that was ultimately diagnosed with pheochromocytoma.

## Case Presentation

This study adhered to the tenets of the Declaration of Helsinki. The patient provided written informed consent for the publication of any potentially identifiable images or data included in this case report.

A 56-year-old man was admitted to the emergency department with acute symptoms of precordia pain, chest tightness and diaphoresis for 1 hour. He had been experiencing these symptoms intermittently since the past 4 years but received no treatment. His medical history included hypertension diagnosed 3 years ago, with the highest recorded systolic pressure of 263 mmHg. He was taking angiotensin-converting enzyme inhibitor (ACEI) to lower his blood pressure (BP), however it was not stable, having frequent ups and downs. In addition, he had a longstanding history of smoking.

**Figure 1:**
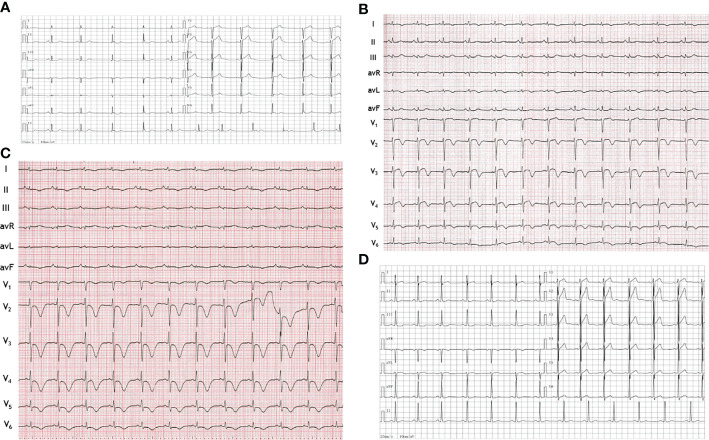
Electrocardiograms. **(A)** Electrocardiogram obtained one month before this admission showed nearly normal. **(B)** Electrocardiogram at hospital admission showed QT prolongation and T-wave inversions in leads I and V1 to V6. **(C)** Electrocardiogram on the third hospital day showed more obvious QT prolongation and T-wave inversions in the limb leads (I, II, III, AVL, AVR and AVF) and the chest leads (V1 to V6). **(D)** Electrocardiogram after surgery showed QT prolongation and T-wave inversions disappeared.

**Figure 2:**
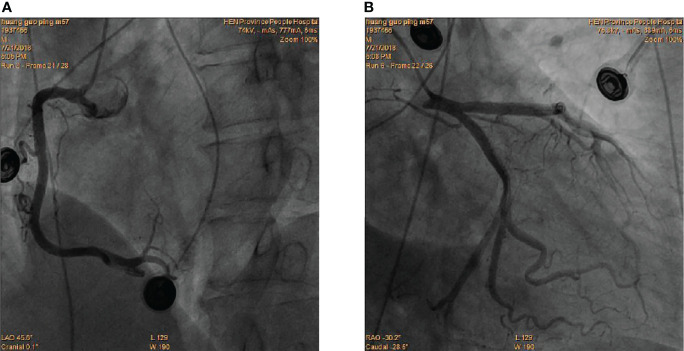
Coronary angiography showed the absence of obstructive coronary lesions in RCA **(A)**, LAD and LCX**(B)**.

At presentation, physical examination revealed stable vital signs with body temperature of 36.6°C, respiratory rate of 16 bpm, heart rate (HR) of 96 bpm, BP of 134/87 mmHg, and normal heart and lung sounds on auscultation. Peripheral edema was also not found. Results of laboratory tests showed elevated troponin-I of 1.18 ng/mL (normal 0 - 0.5 ng/mL), elevated myoglobin (Myo) of 204 ng/ml (normal 0 - 107 ng/mL), and increased white blood cell count (WBC) of 19.1 × 10^9^/L (normal 3.5 - 9.5 × 10^9^/L) with 82.5% neutrophils (normal 40 - 75%) and normal C-reactive protein of 0.1 mg/mL (normal 0–10 mg/mL). Compared with the 12-lead electrocardiogram (ECG) of the last month, this ECG showed QT prolongation (QTc 446 ms) and T-wave inversions in leads I and V1 to V6 (**Figures 1A, B**) . The echocardiogram showed segmental wall motion abnormalities (hypokinesia of inter-ventricular septum, anterior wall, and apex), however left ventricular systolic contractility was preserved (ejection fraction 51%). The patient was highly suspected of acute non-ST segment elevation myocardial infarction (NSTEMI). An urgent coronary angiography (CAG) was performed. However, CAG showed no significant stenosis (**Figures 2A, B**). Next, the patient was transferred to the general ward for further examination and treatment.

**Figure 3:**
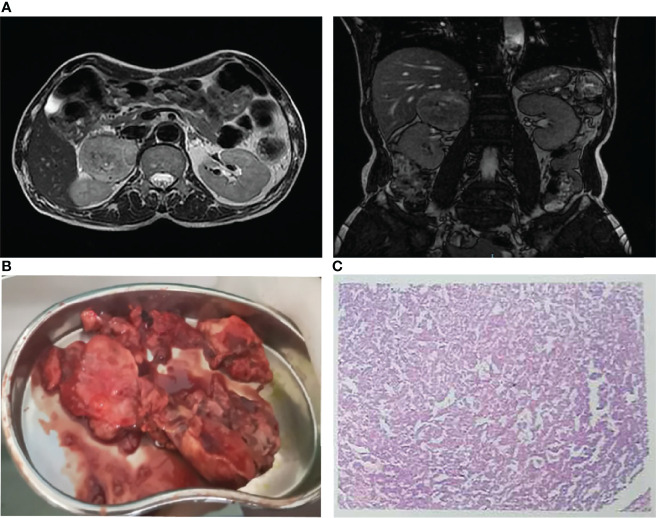
**(A)** Magnetic resonance imaging demonstrated a 68 x 61 mm mass in the right adrenal gland. **(B)** Right adrenalectomy was performed to remove a huge pheochromocytoma. **(C)** Pathological examination showed pheochromocytoma.

On his third hospital day, we stumbled upon a significant BP fluctuation of up to 298/167 mmHg. A repeat ECG showed more obvious QT prolongation (QTc 497 ms) and T-wave inversions in the limb leads (I, II, III, AVL, AVR and AVF) and the chest leads (V1 to V6) (**Figure 1C**). In the context of the lack of findings during CAG, the fluctuating hypertension, together with the apparent ACS and increased WBC prompted the suspicion of pheochromocytoma, which was further supported by the markedly elevated plasma normetanephrine of 2195.91 pg/mL (normal < 90 pg/mL) and plasma metanephrine of 860.22 pg/mL (normal < 180 pg/mL). The magnetic resonance imaging (MRI) revealed a 68 x 61 mm mass in the right adrenal gland (**Figure 3A**). He underwent successful laparoscopic right adrenalectomy, and the pathological findings confirmed adrenal pheochromocytoma (**Figures 3B, C**). After the surgery, the patient was asymptomatic and his BP turned normal gradually. At the outpatient follow-up 1 year later, the postoperative echocardiogram showed resolution of all wall motion abnormalities. His postoperative ECG showed that QT prolongation and T-wave inversions disappeared (**Figure 1D**).

## Discussion

Pheochromocytoma is a rare catecholamine-secreting tumor and is well recognized as “the great mimic” because it can present with a multitude of symptoms, such as hypertension, nausea, fever, flushing, sweating, anxiety, hyperglycaemia and weight loss, as well as arrhythmias, cardiomyopathy, heart failure (4, 5). There is a recently-growing body of literature on a minority of pheochromocytoma patients who present in occasionally ACS, which is bound to mislead physicians into making an incorrect diagnosis.

In the present case, the patient presented with precordia pain and had increased levels of myocardial injury markers, with T-wave inversions on ECG and segmental ventricular wall motion abnormalities on echocardiography. The patient was initially diagnosed with acute NSTEMI. However, CAG showed no acute occlusion of the coronary arteries. Fortunately, the pronounced fluctuation of BP provided us a hint of pheochromocytoma, in which the excess of catecholamine can cause chest discomfort due to an increase in myocardial oxygen consumption and cardiac afterload (6). In addition, high catecholamine levels can induce vasoconstriction of the coronary arteries resulting in elevated troponin level and ECG abnormalities in the absence of coronary occlusion (7). In contrast to ACS, the elevation of troponin level in pheochromocytoma is often mild (8). Limited cases had reported that ECG abnormalities in patients with pheochromocytoma showed ST-segment elevations and/or abnormal Q waves, as well as T-wave inversions and/or QT prolongation (9–12). The prolonged QT interval and T-wave inversions changes were also observed in this case. Previously, Ozyuncu et al. (13) reported the dynamic ECG changes that occurred daily after the pheochromocytoma attack. We also found that the QT prolongation and T-wave inversions on the second day of admission were more significant than that at arrival. These dynamic changes may be attributed to the dynamic changes of catecholamine levels after the initial pheochromocytoma attack.

Although pheochromocytoma can cause multiple cardiovascular complications, most of them are reversible after pheochromocytoma resection. In our case, the patient did not have the typical triad of pheochromocytoma but presented with ACS that caused abnormal changes in ECG and cardiac function. However, the abnormal changes in ECG and echocardiography caused by pheochromocytoma disappeared after tumor resection.

Previous study showed that catecholamine excess in pheochromocytoma was accompanied by an increase in inflammation markers, such as interleukin 6 (IL-6) (14, 15). As a mediator of inflammation, IL-6-induced leukocytosis has been reportedly present in pheochromocytoma. Wang et al. (16) presented a rare case of giant pheochromocytoma with leukemoid reaction. Our patient also showed a significant increase in WBC but normal CRP, which may be associated with pheochromocytoma rather than with concomitant infection.

## Conclusion

Pheochromocytoma can vary widely in the presentation and can mimic many other diseases, which can easily lead to a misdiagnosis. Here, we describe an uncommon presentation of pheochromocytoma mimicking ACS, and the cardiovascular complications are reversible after pheochromocytoma resection. However, this case reminds that clinicians should be familiar with clinical manifestations of pheochromocytoma and must consider pheochromocytoma in the differential diagnosis of patients presenting with ACS without coronary artery occlusion.

## Data Availability Statement

The original contributions presented in the study are included in the article/supplementary material. Further inquiries can be directed to the corresponding author.

## Ethics Statement

The studies involving human participants were reviewed and approved by the Ethics Committee of Henan Provincial People’s Hospital. The patients/participants provided their written informed consent to participate in this study. Written informed consent was obtained from the individual(s) for the publication of any potentially identifiable images or data included in this article.

## Author Contributions

LC was the patient’s physician and collected the data for case presentation. YC conducted the literature search and prepared the first draft of the manuscript. LQ contributed with final manuscript drafting. All authors contributed to the article and approved the submitted version.

## Conflict of Interest

The authors declare that the research was conducted in the absence of any commercial or financial relationships that could be construed as a potential conflict of interest.

## Publisher’s Note

All claims expressed in this article are solely those of the authors and do not necessarily represent those of their affiliated organizations, or those of the publisher, the editors and the reviewers. Any product that may be evaluated in this article, or claim that may be made by its manufacturer, is not guaranteed or endorsed by the publisher.
